# An Estrogen Receptor Dependent Mechanism of Oroxylin A in the Repression of Inflammatory Response

**DOI:** 10.1371/journal.pone.0069555

**Published:** 2013-07-29

**Authors:** Hong Wang, Ying Guo, Xin Zhao, Huiying Li, Guanwei Fan, Haoping Mao, Lin Miao, Xiumei Gao

**Affiliations:** 1 Tianjin State Key Laboratory of Modern Chinese Medicine, Tianjin, China, Key Laboratory of Pharmacology of Traditional Chinese Medical Formulae, Ministry of Education, Tianjin University of Traditional Chinese Medicine, Tianjin, China; 2 Tianjin Key Laboratory of Traditional Chinese Medicine Pharmacology, Tianjin, China; 3 Institute of Traditional Chinese Medicine, Tianjin University of Traditional Chinese Medicine, Tianjin, China; Northwestern University, United States of America

## Abstract

Oroxylin A, a natural flavonoid, is one of the main bioactive compounds that underlie the anti-inflammatory effect of the medicinal herb *Scutellaria* *baicalensis* Georgi widely used in southeastern Asia; however, the molecular mechanisms for the therapeutic benefits remain largely unclear. In this study, we found that Oroxylin A induces estrogen-responsive gene expression and promoter activity. In macrophages, Oroxylin A treatment significantly attenuates lipopolysaccharide (LPS)-induced but not basal inflammatory response, including nitric oxide (NO) production and the expression of inflammatory mediators (i.e., iNOS and COX-2) and cytokines (i.e., TNF-α, IL-1β, and IL-6), in an estrogen receptor (ER)-dependent manner. Oroxylin A treatment also dramatically decreases LPS-induced secretion of pro-inflammatory cytokines. Furthermore, the downregulation of all these inflammatory parameters by Oroxylin A was abolished when cells were pretreated with specific ER antagonist. Thus, Oroxylin A is a novel phytoestrogen and exhibits anti-inflammatory effects that are mediated by ER activity.

## Introduction

Oroxylin A (C_16_H_12_O_5_, Figure S1) is one of the main bioactive compounds purified from the root of the medicinal herb *Scutellaria* *baicalensis* Georgi that has been widely used in China, Japan, and Korea for treating inflammation and infections in the respiratory and gastrointestinal system [1]. Although many biological activities of Oroxylin A, including anti-tumor [2,3], anti-bacterial [4], and cognitive enhancement [5], have been reported, the underlying molecular mechanisms are largely unknown.

Oroxylin A is a natural flavonoid, and flavonoids are a class of polyphenolic compounds. Studies from our group and others have showed that some flavonoids have estrogenic activity [6–10] and thus, are named phytoestrogens. Whether Oroxylin A is also a phytoestrogen remains to be determined.

The effects of estrogen and phytoestrogens are mediated through two well-characterized intracellular receptors, estrogen receptor (ER) α and β [11,12]. ERs are members of the nuclear receptor superfamily and act as a ligand-activated transcription factors to regulate the expression of target genes. They are expressed in various immune cells, including macrophages [13,14] that play a critical role in many inflammatory diseases by expressing pro-inflammatory mediators, including tumor necrosis factor (TNF)-α, Interleukin (IL)-1, interleukin (IL)-6, cyclooxygenase-2 (COX-2), and inducible nitric oxide synthase (iNOS) [15,16]. Among various inflammatory stimuli, lipopolysaccharide (LPS) is most frequently used to study macrophage biology [17].

In this study, we have evaluated the estrogenic activity of Oroxylin A and investigated its anti-inflammatory properties by using an *in vitro* model system where the inflammatory response is induced in RAW 264.7 macrophages by LPS treatment [18]. Our results suggest that Oroxylin A is a new phytoestrogen, and it activates the expression of estrogen target genes and potently attenuates LPS-induced expression of a panel of pro-inflammatory mediators through the activity of ERs.

## Materials and Methods

### Reagents

Oroxylin A was purchased from the Chinese Institute for Drug and Biological Product Control (Beijing, China). Charcoal dextran–stripped FBS (CD–FBS) was purchased from Biological Industries (Kibbutz Beit Haeme of Israel). Specific ER antagonist ICI 182,780 was purchased from Tocris Bioscience (Ellisville, MO, USA). IL-1β, IL-6, and TNF-α enzyme-linked immunosorbent assay (ELISA) kits were obtained from R&D Systems (Minneapolis, MN, USA). Griess Reagent System was purchased from Beyotime (Nanjin, Jiangsu, China). TaqMan Reverse Transcription Reagents and SYBR Green PCR Master Mix reagent kit were obtained from Applied Biosystems (Foster City, CA, USA). Nuclear Extraction kit was purchased from Millipore (Billerica, MA, USA). 17β-estradiol (E_2_), ICI 182,780, and Oroxylin A were dissolved in DMSO and further diluted in cell culture media so that the final DMSO concentration did not exceed 0.1% v/v. All other reagents used in cell culture and transfection were obtained from Sigma-Aldrich (St. Louis, MO, USA).

### Cell Culture

HeLa, MCF-7, and RAW264.7 cells were obtained from American Type Culture Collection (ATCC). Cells were maintained in high glucose Dulbecco’s Modified Eagle Medium (DMEM) supplemented with 10% FBS and penicillin and streptomycin in a 37°C humidified incubator containing 5% CO_2_.

### Transient transfection and reporter assay

Mammalian expression vectors ERα and ERβ were gifts from Dr. R.H. Karas (Tufts Medical Center, Boston, USA). The luciferase reporter plasmid carrying 3× vitellogenin ERE was kindly provided by Dr. J. Zhang (Nankai University, Tianjin, China). Cells were plated in triplicate in 24-well plates at a density of 2×10^5^ cells/well in 10% CD–FBS. After attachment and growth for 24 h, the cells were co-transfected with the reporter plasmid ERE-TK-Luc, ERα/βexpression plasmids, and pRL-TK control plasmid, which contains a Renilla luciferase gene to allow for normalizing transfection efficiency. Transfection was performed in serum-free, antibiotic-free DMEM media using Lipofectamine 2000 (Invitrogen/Life Technologies, Carlsbad, CA) according to manufacturer’s instructions. Then, the cells were treated with individual test compounds for 24 h and lysed. Aliquots from each well were divided into two 96-well plates for luciferase and renillia activity determination using a luminescence counter (Flexstation 3 Molecular Devices Company, CA, USA). Experiments were performed at least three times and the data were assessed as units of firefly luciferase activities normalized to the renilla luciferase control activities from individual wells.

### RNA isolation and quantitative real time RT-PCR (qRT-PCR)

For assessments of pS2 mRNA expression, MCF-7 cells were seeded into 6-well plates at a density of 5×10^5^ cells per well and incubated with phenol red-free DMEM supplemented with 3% CD–FBS and maintained in 5% CO_2_ in air at 37°C for overnight. Then, test compounds were added to the medium and 10 h later, the medium was removed and the cells were scraped from the dish for RNA extraction. The total RNA was purified with TRIzol reagent (GIBCO) according to the manufacturer’s protocol. The RNA samples were treated with DNase I, and cDNA was made from each sample. cDNAs of the pS2 and an internal reference gene (GAPDH) were quantified using a fluorescence-based real time detection method (SYBR Green PCR Master Mix reagent kits, Applied Biosystems, Foster City, USA), which was performed with the ABI PRISM 7300 Sequence Detection System. The sequences of primers for amplification of human pS2 are reported in Table S1. Data were analyzed by using the comparative threshold cycle (Ct) method. Ct values from the genes of interest were normalized with the values from corresponding GAPDH reactions.

For assessments of the mRNA expression of pro-inflammatory mediators, the RAW264.7 cells were grown in 6-well plates in phenol red-free DMEM with 10% CD–FBS. After approximately 80% confluence, cells were pretreated with Oroxylin A for 2 h and then stimulated with LPS (1µg/ml) for an additional 8 h. Total RNA extracted from RAW264.7 cells by using the Trizol reagent (Invitrogen) was reverse-transcribed into cDNA with TaqMan Reverse Transcription Reagents. Real time PCR was performed using SYBR Green PCR Master Mix reagent kits (Applied Biosystems) and the specific primers (Table S1).

### Measurement of nitric oxide (NO) in the cell culture medium

The RAW264.7 cells were plated in 48-well plates at a density of 2x10^6^ cells per well in phenol red-free DMEM with 10% CD–FBS for 24 h. After pretreatment with Oroxylin A for 2 h the cells were stimulated with LPS (1µg/ml) in serum-free DMEM for an additional 18 h. The supernatant from the cultured cells was collected and centrifuged to remove cell debris and transferred to 96-well plates, and then reacted using a NO detection kit (Beyotime, China). Values were calculated by measuring the absorbance at 540 nm using a plate reader.

### Enzyme-linked immunosorbent assay (ELISA)

One day after seeding in 48-well plates in phenol red-free DMEM, the RAW264.7 cells were treated with Oroxylin A and 2 h later, stimulated with LPS (1µg/ml) for additional 18 h. Then the medium was collected from each well and centrifuged and the supernatant was analyzed by using special ELISA kits for IL-1β, IL-6 and TNFα(R&D systems Inc, Minneapolis, MN) as directed by the manufacturer’s instructions.

### Statistical analysis

The results are expressed as mean ± S.D. ANOVA followed by a post hoc multiple comparison was performed. Dunnett’s test was used to compare increasing doses of the test compounds with the respective control. A *P*-value less than 0.05 is considered significant.

## Results

### Oroxylin A induces estrogen-responsive gene expression and promoter activity through estrogen receptor (ER) activity

**Figure 1:**
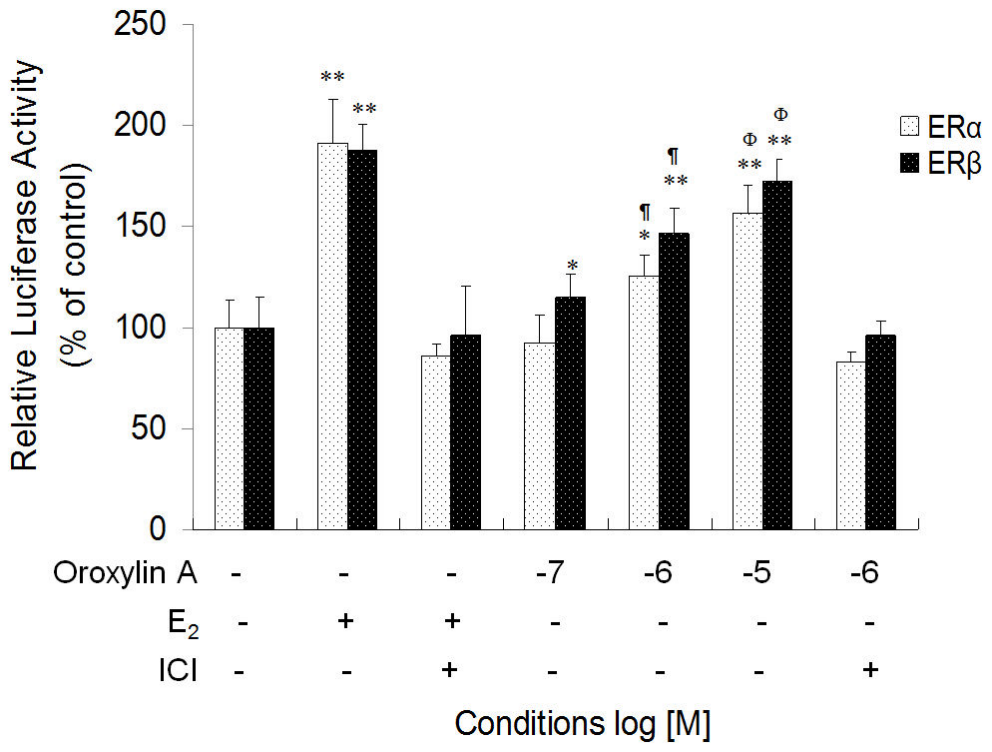
Oroxylin A activates the transcriptional activity of ERα and ERβ. HeLa cells were co-transfected with pERE-luc, pRL-TK, and a plasmid coding for ERα or ERβ and 18 h later, treated with Vehicle (-), Estrogen (E_2_; 10^-8^ M), Oroxylin A (10^-7^–10^-5^M), and/or ICI 182,780 (10^-7^ M) for an additional 24 h. Then, ER activities (i.e., Luciferase activities) of different treatment groups were measured and presented as fold increase relative to the levels in cells treated with Vehicle only. n=3 per group; *p<0.05, **p<0.01 vs. Vehicle controls. ^¶^P < 0.05 vs. cells treated with Oroxylin A (10^-7^ M); ^Φ^ P < 0.05 vs. cells treated with Oroxylin A (10^-6^ M). Shown is representative of three independent experiments with similar results.

We initiated our investigation by assessing whether Oroxylin A displays estrogenic activity. ERE-dependent promoter-luciferase reporter assays were performed in HeLa cells that had been transfected with plasmids coding for ERα or ERβ and then treated with estradiol (E_2_) or Oroxylin A (at concentrations from 10^-7^ ~10^-5^ M). Oroxylin A treatment increased the activity of both ERα and ERβ in a concentration-dependent manner (Figure 1).

**Figure 2:**
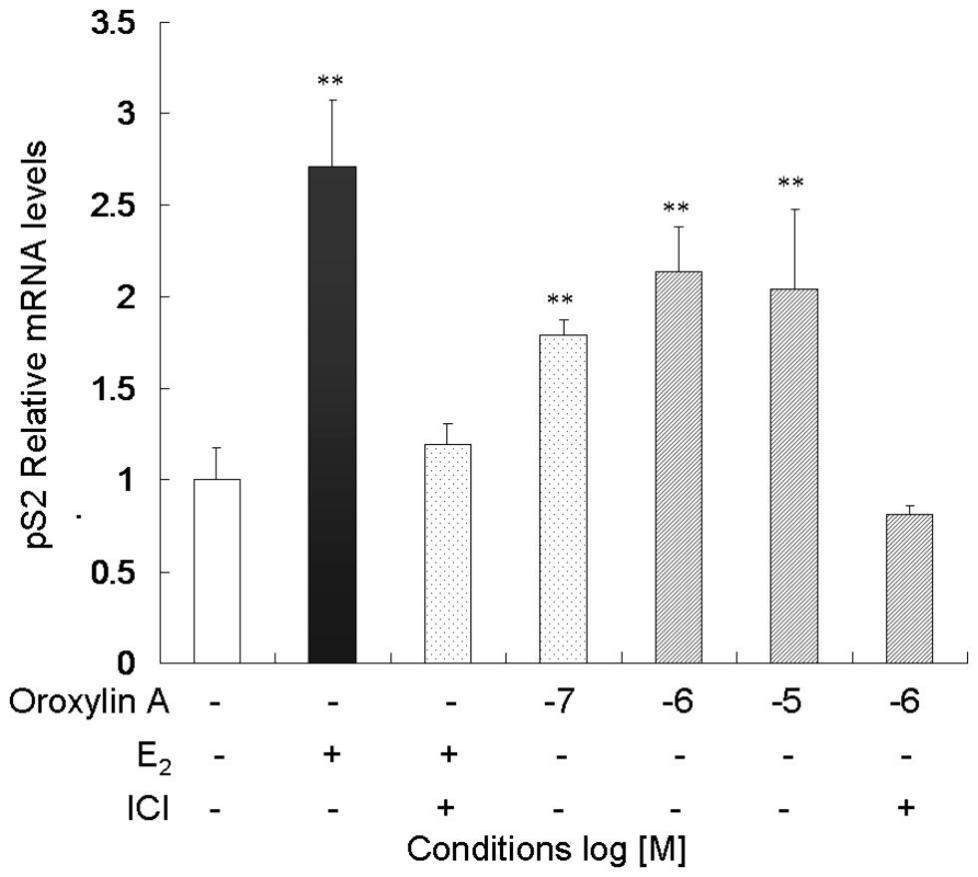
Oroxylin A induces mRNA expression of endogenous estrogen target gene pS2. MCF-7 cells were incubated for 10 h in the presence of Vehicle (-), E_2_ (10^-8^ M), ICI 182,780 (10^-7^ M), and/or Oroxylin A at indicated concentrations and then subjected to qRT-PCR analyses. N=3 per treatment; **p<0.01 vs. Vehicle (-). Shown is representative of three independent experiments with similar results.

Next, we evaluated whether Oroxylin A activates the expression of endogenous estrogen target genes. MCF-7 cells were treated with E_2_ and serial concentrations of Oroxylin A for 10 h and then, analyzed for the mRNA expression of pS2 gene by qRT-PCR. Oroxylin A induced a marked increase in pS2 mRNA expression, which peaked 2.2 folds-of-induction (Figure 2), while the cell viability was unaffected (Figure **S2**). Notably, the induction of both pS2 mRNA expression and ERE promoter activity were abolished when the cells were co-treated with specific ER antagonist ICI 182,780 (Figures **1** and 2). These results suggest that Oroxylin A can induce transcription of estrogen target genes through the action of ER. Thus, Oroxylin A is an estrogen mimetic or a “phytoestrogen”.

### Oroxylin A treatment attenuates LPS-induced iNOS and COX-2 expression and the production of nitric oxide (NO) in macrophages in an ER-dependent manner

**Figure 3:**
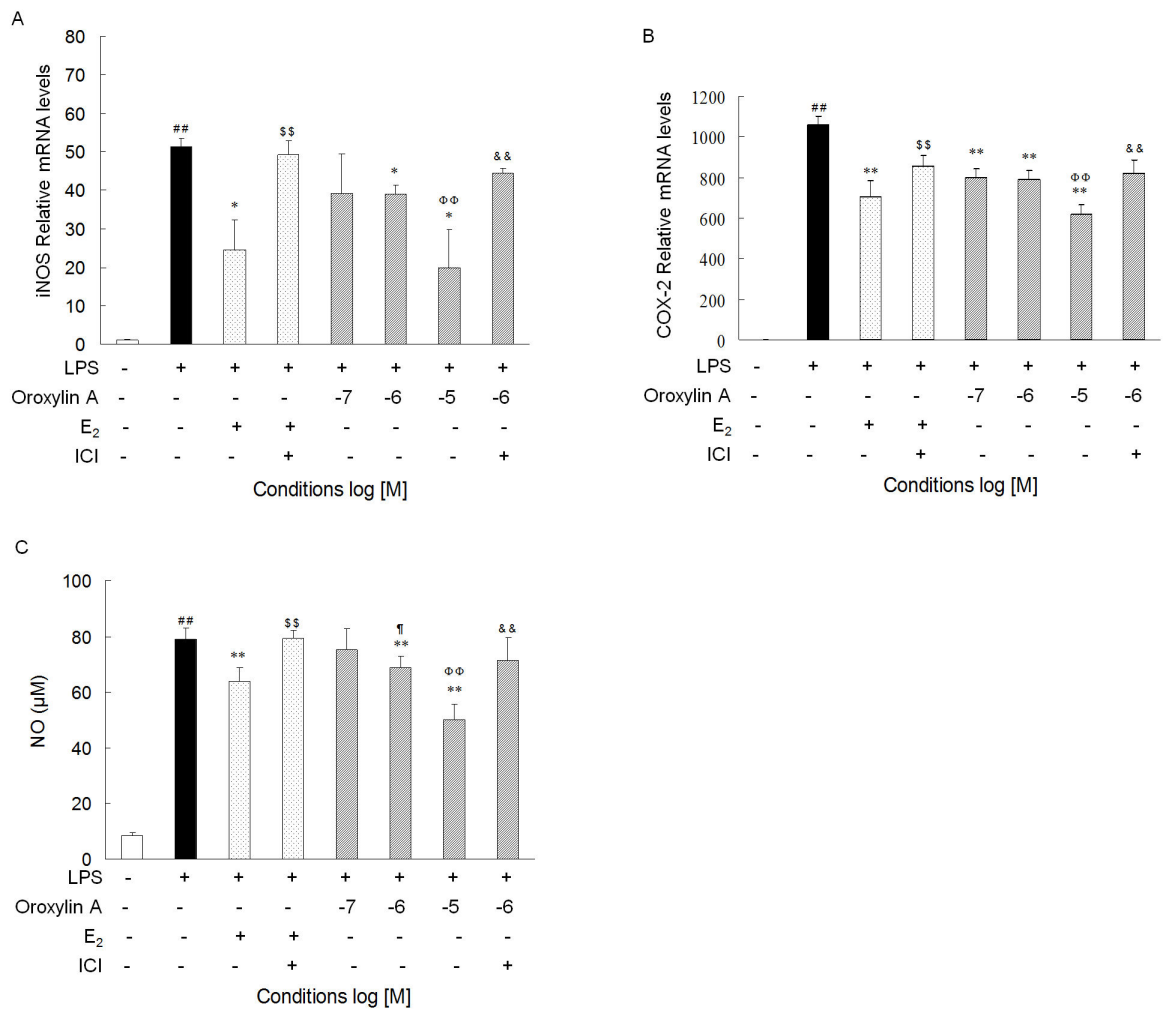
Oroxylin A suppresses LPS-induced iNOS and COX-2 mRNA expression and the production of NO in ER-dependent manner. (**A**–**B**) RAW 264.7 cells were pre-incubated with Oroxylin A at indicated concentrations, E_2_ (10^-8^ M), and/or ICI 182,780 (10^-7^ M) for 2 h, then LPS was added (1µg/ml), and the cells were cultured for an additional 8 h. The iNOS and COX-2 gene expression was analyzed by qRT-PCR. N=3; *P < 0.05, **P < 0.01 vs. cells treated with LPS only; ^# #^ P < 0.01 vs. Vehicle; ^$ $^ P < 0.01 vs. cells treated with LPS plus E_2_ (10^-8^ M); ^& &^ P < 0.01 vs. cells treated with LPS plus Oroxylin A in the same concentration. (**C**) RAW 264.7 cells were treated with LPS (1 µg/ml), Oroxylin A, E_2_ (10^-8^ M), and/or ICI 182,780 (10^-7^ M) for 18 h, and the amount of nitrite in the supernatant of each treatment group was quantified using Griess reagent. N=5; **P < 0.01 vs. cells treated with LPS only; ^# #^ P < 0.01 vs. Vehicle; ^$ $^ P < 0.01 vs. cells treated with LPS plus E_2_ (10^-8^ M); ^& &^ P < 0.01 vs. cells treated with LPS plus Oroxylin A in the same concentration. ^¶^P < 0.05 vs. cells treated with LPS plus Oroxylin A (10^-7^ M); ^ΦΦ^ P < 0.01 vs. cells treated with LPS plus Oroxylin A (10^-6^ M). Shown are representatives of three independent experiments with similar results.

Because estrogen plays an important role in the regulation of inflammation, we investigated whether Oroxylin A modulates LPS-induced inflammatory response in macrophages. RAW264.7 cells were incubated with Oroxylin A, E_2_, or vehicle for 2 h; then LPS was added, and the cells were culture for additional 8 h before assessments of iNOS and COX-2 expression. LPS treatment led to a marked increase in the levels of iNOS and COX-2 mRNAs (Figure 3A-B). Pre-treatment with Oroxylin A and E_2_, but not with vehicle, significantly attenuated LPS induction of iNOS and COX-2 expression. However, the repressor effects of Oroxylin A and E_2_ was largely abrogated when the cells were co-treated with ICI 182,780 (Figure 3A-B).

Because iNOS catalyzes the generation of NO, we measured NO in the culture media of cells that had been treated with LPS for 18 h with or without pre-treatment with Oroxylin A or E2. Consistently, we found that NO was markedly induced by LPS treatment, that the LPS induction of NO is repressed by E_2_ and Oroxylin A in a concentration-dependent manner, and that the repressor effects of E_2_ and Oroxylin A on LPS induction of NO were abolished by co-treatment of cells with ICI 182,780 (Figure 3C). Collectively, these results suggest that Oroxylin A represses LPS-induced expression of inflammatory mediators, and this repressor effect of Oroxylin A is mediated by ER activity. Of note, neither a cytotoxic effect (CCK assay) nor an alteration of cell viability (MTT assay) was observed in RAW264.7 cells treated with Oroxylin A at the experimental concentrations (i.e., 10^-7^M, 10^-6^M, and 10^-5^M) (data not shown).

### Oroxylin A treatment suppresses LPS-induced secretion of pro-inflammatory Cytokines in an ER-dependent manner

**Figure 4:**
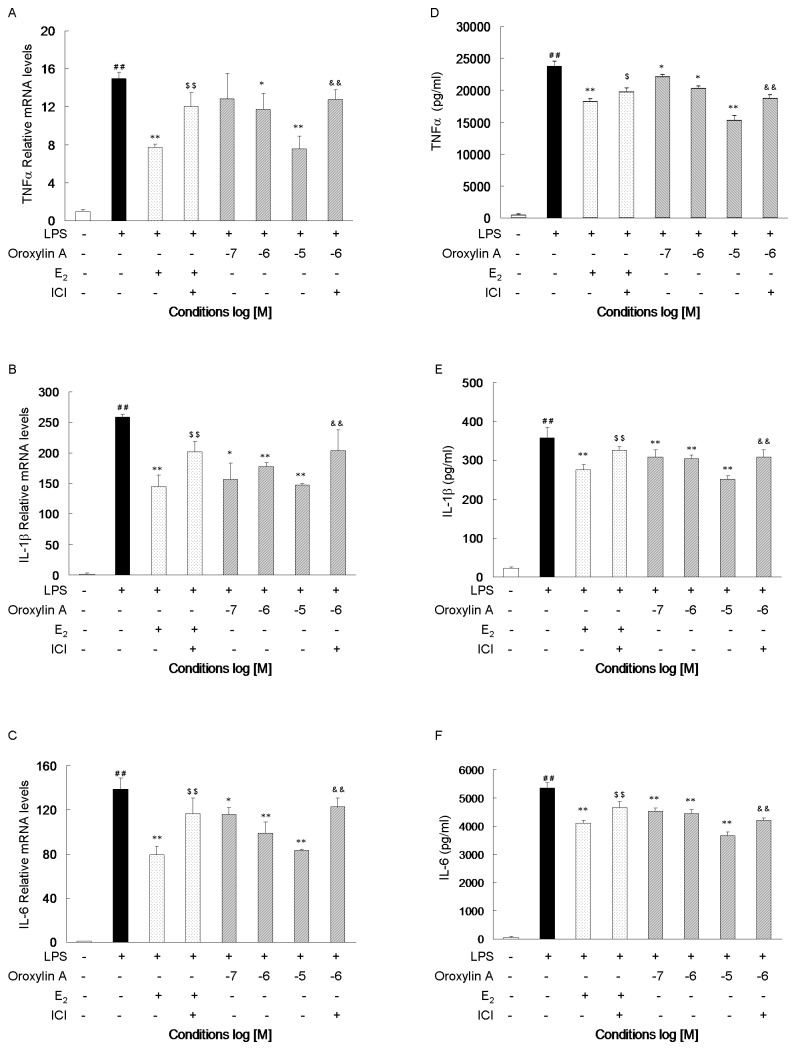
Oroxylin A suppresses LPS-induced mRNA expression and secretion of TNF-α, IL-1β, and IL-6 in ER-dependent manner. (**A**–**C**) RAW 264.7 cells were incubated with Oroxylin A, E_2_ (10^-8^ M), and/or ICI 182,780 (10^-7^ M) for 2 h, then LPS was added (1µg/ml), and the cells were cultured for an additional 6 h before qRT-PCR analyses. (**D**–**F**) RAW 264.7 cells were cultured with LPS (1µg/ml), Oroxylin A, E_2_ (10^-8^ M), and/or ICI 182,780 (10^-7^ M) for 18 h and then, the amount of cytokines in the supernatant were quantified by ELISA. N=3; *P < 0.05**P < 0.01 vs. cells treated with LPS only; ^# #^ P < 0.01 vs. Vehicle; ^$^ P < 0.05, ^$ $^ P < 0.01 vs. cells treated with LPS plus E_2_ (10^-8^ M); ^& &^ P < 0.01 vs. cells treated with LPS plus Oroxylin A in the same concentration. Shown are representatives of three independent experiments with similar results.

Because TNF-α, IL-1β and IL-6 play a critical role in the inflammatory process, we investigated the effect of Oroxylin A on their expression. RAW264.7 cells were treated for 6 h and 18 h with or without co-treatment with Oroxylin A, E_2_, or ICI, and then analyzed for mRNA expression (qRT-PCR) and protein secretion (ELISA), respectively (Figure 4). Consistent with our observations with iNOS, COX-2, and NO, treatment of cells with Oroxylin A suppressed the LPS-induced mRNA expression and secretion of TNF-α, IL-1β, and IL-6 in a concentration-dependent manner (Figure 4). Furthermore, the Oroxylin A-mediated repression of mRNA expression and protein secretion were largely abolished by ICI co-treatment.

## Discussion

In this study, we found that Oroxylin A is a new phytoestrogen that activates estrogen-responsive promoter activity and the expression of endogenous estrogen target genes. Our results also indicate that Oroxylin A displays a potent anti-inflammatory activity, attenuating LPS-induced expression of pro-inflammatory mediators and cytokines in macrophages. The anti-inflammatory effects and ER transcriptional activity of Oroxylin A occur at similar concentrations, and importantly all these Oroxylin A functions appear to be mediated by ER activity.

Macrophages are recognized as an important source of inflammatory factors including NO, iNOS, COX-2, TNF-α, IL-1β and IL-6, and therefore, represent a cellular target for the prevention, control, and cure of inflammatory damages [17,19–21]. We observed that the anti-inflammatory effects of Oroxylin in macrophages are abolished by co-administration of a specific ER antagonist, suggesting that activation of ER is a necessary step in mediating the inflammatory proteins synthesis. These findings are in agreement with previous observations made by us and other groups, which demonstrated that estrogen decreases production of inflammatory cytokines and inhibits macrophage infiltration in damaged tissues [22–27]. Interestingly, some other flavonoids have also been shown to inhibit NO production in response to inflammatory stimuli [28–31]. Collectively, our data and the results from other labs suggest that ERs in macrophage could serve a molecular target for reducing inflammation.

A main finding of our study is that Oroxylin A activates ERs. Such a function is also supported by its polyphenolic structures. Nevertheless, the domains mediating the interaction between Oroxylin A and ERs and other potential associating proteins are yet to be identified. An outstanding question is whether Oroxylin A-induced ER activation directly down-regulates the transcription of those pro-inflammatory genes. This is intriguing particularly for iNOS and COX-2 since the promoters of both genes do not appear to contain a recognizable ERE. One explanation is that iNOS and COX-2 may be regulated indirectly by other Oroxylin A-induced genes. Alternatively, the activated ERs may interact and form complex with other transcription factors, such as NF-κB, AP-1, and STATs and prevent them from binding to their cognate response elements [32,33]. Clearly, these hypotheses need to be tested in future studies.

In conclusion, Oroxylin A possesses estrogenic activity and inhibits LPS-elicited expression of pro-inflammatory mediators and cytokines through ER activity. In the last decade, there has been an increasing interest in the use of natural products to modulate inflammatory disorders because of their lesser side effects and cytotoxicity. The observations made in this study suggest the mode of actions of phytoestrogenic compounds in preventing inflammation, which may be helpful in developing new therapeutics for the treatment of inflammatory disease, especially in the population of aging women with low level of estrogen.

## Supporting Information

Figure S1 The structure of Oroxylin A. (TIF)

Figure S2 Oroxylin A treatment does not affect cell viability. MCF-7 cells were incubated in the presence of Vehicle (-) and Oroxylin at concentrations from 10^-7^-10^-5^ M for 24 h and then, cell viability was assessed with MTT assay. N=3; NS, not significant.
(TIF)

Table S1 Primers for real-time PCR. (DOC)
