# Supplementary figures and images for: An Estrogen Receptor Dependent Mechanism of Oroxylin A in the Repression of Inflammatory Response
Source: PLoS One. 2013 Jul 29;8(7):e69555. doi: 10.1371/journal.pone.0069555 (PMC3726624; doi:10.1371/journal.pone.0069555)

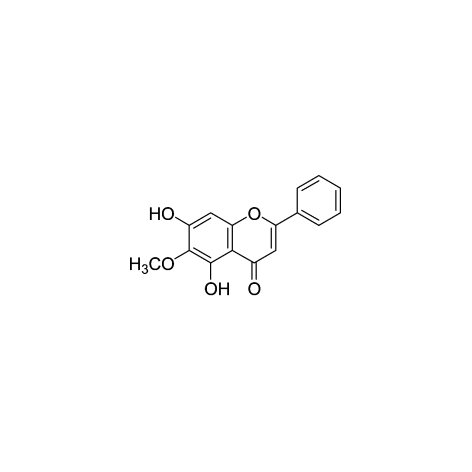

Supplement: Figure S1 — (TIF) [file pone.0069555.s001.tif]

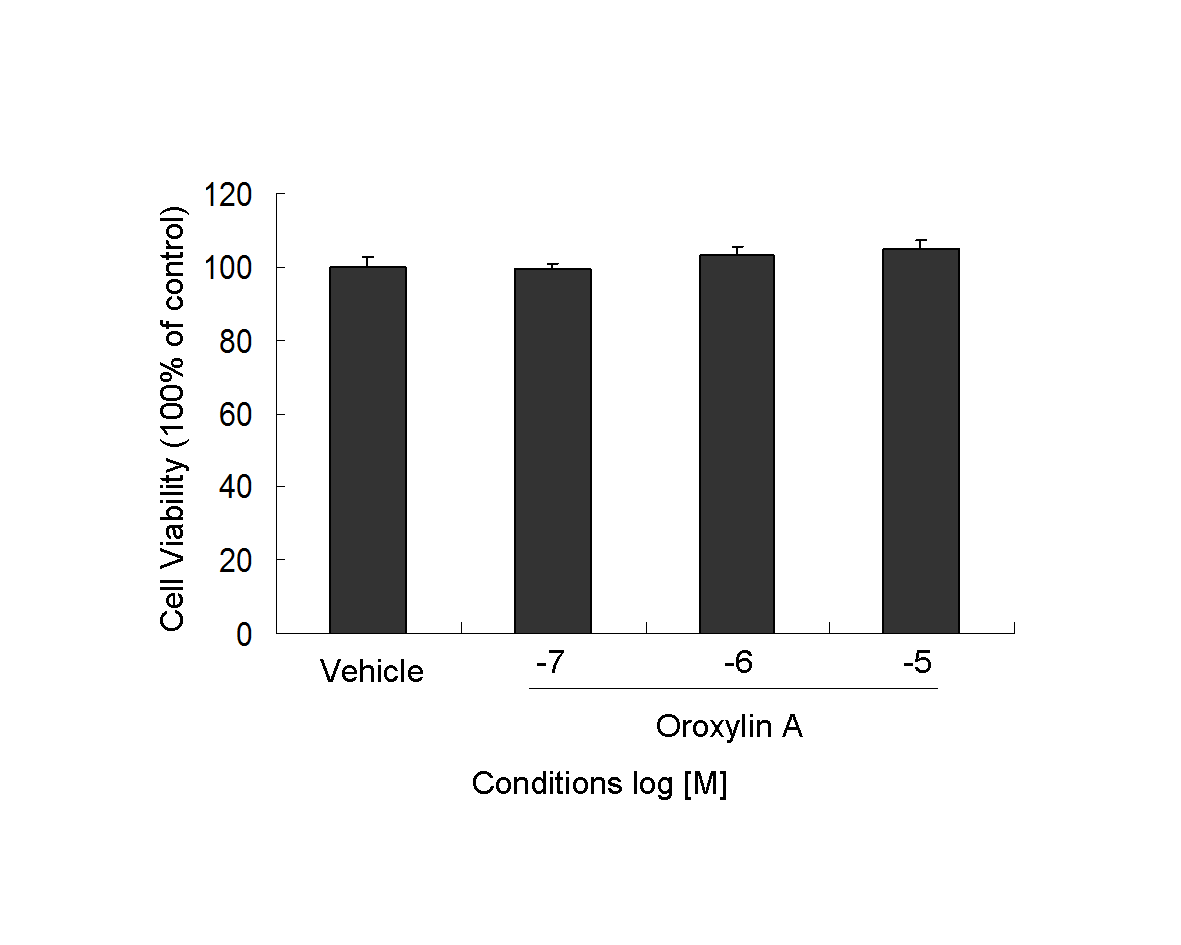

Supplement: Figure S2 — MCF-7 cells were incubated in the presence of Vehicle (-) and Oroxylin at concentrations from 10-7-10-5 M for 24 h and then, cell viability was assessed with MTT assay. N=3; NS, not significant. (TIF) [file pone.0069555.s002.tif]
