# Supplementary material for: An Estrogen Receptor Dependent Mechanism of Oroxylin A in the Repression of Inflammatory Response
Source: PLoS One. 2013 Jul 29;8(7):e69555. doi: 10.1371/journal.pone.0069555 (PMC3726624; doi:10.1371/journal.pone.0069555)
Supplement: Table S1 — (DOC) [file pone.0069555.s003.doc]

**Table S1. Primers for real-time PCR**

| Gene | Primer pair (5'–3')  F, forward; R, reverse |
| --- | --- |
| Human pS2  Human GAPDH  Murine TNF-α | F: GGCCCAGACAGAGACGTGTA  R: GAGGGACGTCGATGGTATTAGG  F: ACCCACTCCTCCACCTTTG  R: CTCTTGTGCTCTTGCTGGG  F: TTCTGTCTACTGAACTTCGGGGTGATCGGTCC |
|  | R: GTATGAGATAGCAAATCGGATGACGGTGTGGG |
| Murine COX-2 | F: TGAGTACCGCAAACGCTTCTC |
|  | R: TGGACGAGGTTTTTCCACCAG |
| Murine iNOS | F: GGCAGCCTGTGAGACCTTTG |
|  | R:GCATTGGAAGTGAAGCGTTTC |
| Murine IL-1β | F:CGCAGCAGCACATCAACAAGAGC |
|  | R:TGTCCTCATCCTGGAAGGTCCACG |
| Murine IL-6 | F:TCCAGTTGCCTTCTTGGGAC |
|  | R:GTGTAATTAAGCCTCCGACTTG |
| Murine β-action | F:AGAGGGAAATCGTGCGTGAC |
|  | R:CAATAGTGATGACCTGGCCGT |
